# Supplementary material for: Cytosolic fructose - an underestimated player in the regulation of sucrose biosynthesis
Source: BMC Plant Biol. 2025 Apr 25;25:535. doi: 10.1186/s12870-025-06493-y (PMC12032757; doi:10.1186/s12870-025-06493-y)
Supplement: Supplementary file 1 — Supplementary Material 1: Fig. S1: Λ matrix of the model. Fig. S2: Θ matrix of the model. Fig. S3: Stability analysis overview of different F flux sizes and external sucrose concentrations. Fig. S4: Histograms of Eigenvalue real part maxima. Fig. S5: Influence of additional weak hexokinase inhibitions to prior regulations on the cytosolic sucrose cycle stability. Fig. S6: Influence of single activated sucrose-phosphate synthase (SPS) by phosphorylated sugars on the cytosolic sucrose cycle stability. Supp. Table 1: Calculated subcellular concentrations of metabolites in Ler and gin2-1 for all here tested conditions. Supp. Table 2: Proportions of positive maximal Eigenvalues for all tested regulations. https://git.rwth-aachen.de/Lisa.Fuertauer/skm-sucrose-cycling. GitLab Project ID 107389. [file 12870_2025_6493_MOESM1_ESM.zip › Revised Supplementary Figures_Giesbrecht et al.pdf]

Cytosolic fructose - an underestimated player in the regulation of sucrose biosynthesis

Oliver Giesbrecht[1], Christina Bonn[1], and Lisa Fürtauer[1,*]

[1]Plant Molecular Systems Biology, Department of Biology III, RWTH Aachen University, 52074 Aachen, Germany

[*]Lisa.Fuertauer@bio3.rwth-aachen.de

ORCID IDs:

Oliver Giesbrecht: Christina Bonn: Lisa Fürtauer:

1 Supplementary information

$$\Lambda = \begin{pmatrix}
\frac{\alpha F'}{[A]} & -\frac{\beta\alpha F'}{[A]} & 0 & 0 & 0 & 0 & 0 & 0 & 0 & -\frac{(1-\beta)\alpha F'}{[A]} & 0 & 0 & 0 & 0 \\
0 & \frac{\beta\alpha F'}{[B]} & \frac{-F'}{[B]} & 0 & 0 & 0 & 0 & 0 & \frac{0.5\,(1-\beta\alpha)F'}{[B]} & 0 & 0 & 0 & \frac{0.5\,(1-\beta\alpha)F'}{[B]} & 0 \\
0 & 0 & \frac{F'}{[C]} & -\frac{\beta\alpha F'}{[C]} & -\frac{\gamma(1-\beta\alpha)F'}{[C]} & 0 & -\frac{(1-\gamma)(1-\beta\alpha)F'}{[C]} & 0 & 0 & 0 & 0 & 0 & 0 & 0 \\
0 & 0 & 0 & 0 & 0 & 0 & \frac{0.5(1-\gamma)(1-\beta\alpha)F'}{[D]} & \frac{0.5\gamma(1-\beta\alpha)F'}{[D]} & -\frac{0.5(1-\beta\alpha)F'}{[D]} & 0 & 0 & 0 & 0 & 0 \\
0 & 0 & 0 & 0 & \frac{\gamma(1-\beta\alpha)F'}{[E]} & -\frac{\gamma(1-\beta\alpha)F'}{[E]} & 0 & 0 & 0 & 0 & 0 & 0 & 0 & 0 \\
0 & 0 & 0 & 0 & 0 & \frac{0.5\gamma(1-\beta\alpha)F'}{[F]} & 0 & -\frac{0.5\gamma(1-\beta\alpha)F'}{[F]} & 0 & 0 & 0 & 0 & 0 & 0 \\
0 & 0 & 0 & 0 & 0 & 0 & 0 & 0 & 0 & \frac{(1-\beta)\alpha F'}{[G]} & 0 & 0 & 0 & -\frac{(1-\beta)\alpha F'}{[G]} \\
0 & 0 & 0 & 0 & 0 & 0 & \frac{0.5(1-\gamma)(1-\beta\alpha)F'}{[H]} & 0 & 0 & 0 & 0 & \frac{0.5\gamma(1-\beta\alpha)F'}{[H]} & -\frac{0.5(1-\beta\alpha)F'}{[H]} & 0 \\
0 & 0 & 0 & 0 & 0 & \frac{0.5\gamma(1-\beta\alpha)F'}{[I]} & 0 & 0 & 0 & 0 & 0 & -\frac{0.5\gamma(1-\beta\alpha)F'}{[I]} & 0 & 0 \\
0 & 0 & 0 & \frac{\beta\alpha F'}{[Suc_{ext}]} & 0 & 0 & 0 & 0 & 0 & 0 & -\frac{\beta\alpha F'}{[Suc_{ext}]} & 0 & 0 & 0
\end{pmatrix}$$

Figure S1: Λ **matrix of the model.** The matrix consists of 10 metabolite pools (rows): A := P-$Sug_{plastidic}$, B := P-$Sug_{cytosolic}$, C := $Suc_{cytosolic}$, D := $Glc_{cytosolic}$, E := $Suc_{vacuolar}$, F := $Glc_{vacuolar}$, G := $starch_{plastidic}$, H := $Frc_{cytosolic}$, I := $Frc_{vacuolar}$ and 14 fluxes (columns). The concentrations depicted in the denominator are taken from the metabolic dataset. (α), (β) and (γ) were randomized between 0 and 1 for every simulation run. F was set to 1, but also F = 2 and F = 10 were initially tested (Fig S4).

$$
\Theta = \begin{pmatrix}
0 & 0 & 0 & 0 & 0 & 0 & 0 & 0 & 0 & 0 \\
\theta_{2,1} & 0 & 0 & 0 & 0 & 0 & 0 & 0 & 0 & 0 \\
0 & \theta_{3,2} & 0 & \theta_{3,4} & 0 & 0 & 0 & \theta_{3,8} & 0 & 0 \\
0 & 0 & \theta_{4,3} & 0 & 0 & 0 & 0 & 0 & 0 & 0 \\
0 & 0 & \theta_{5,3} & 0 & 0 & \theta_{5,6} & 0 & 0 & \theta_{5,9} & 0 \\
0 & 0 & 0 & 0 & \theta_{6,5} & \theta_{6,6} & 0 & 0 & \theta_{6,9} & 0 \\
0 & 0 & \theta_{7,3} & \theta_{7,4} & 0 & 0 & 0 & \theta_{7,8} & 0 & 0 \\
0 & 0 & 0 & 0 & 0 & \theta_{8,6} & 0 & 0 & 0 & 0 \\
0 & \theta_{9,2} & 0 & \theta_{9,4} & 0 & 0 & 0 & 0 & 0 & 0 \\
\theta_{10,1} & 0 & 0 & 0 & 0 & 0 & 0 & 0 & 0 & 0 \\
0 & 0 & 0 & 0 & 0 & 0 & 0 & 0 & 0 & \theta_{11,10} \\
0 & 0 & 0 & 0 & 0 & 0 & 0 & 0 & \theta_{12,9} & 0 \\
0 & \theta_{13,2} & 0 & 0 & 0 & 0 & 0 & \theta_{13,8} & 0 & 0 \\
0 & 0 & 0 & 0 & 0 & 0 & \theta_{14,7} & 0 & 0 & 0
\end{pmatrix}
$$

Figure S2: Θ **matrix of the model.** The matrix consists of 14 influenced fluxes (rows) and 10 influencing metabolites (columns). The matrix depicts all consistent elasticities (black, randomized between 0 and 1) , all implemented activations (green, set to 1 or 0.33) and all implemented inhibitions (red, set between 0 and -0.99).

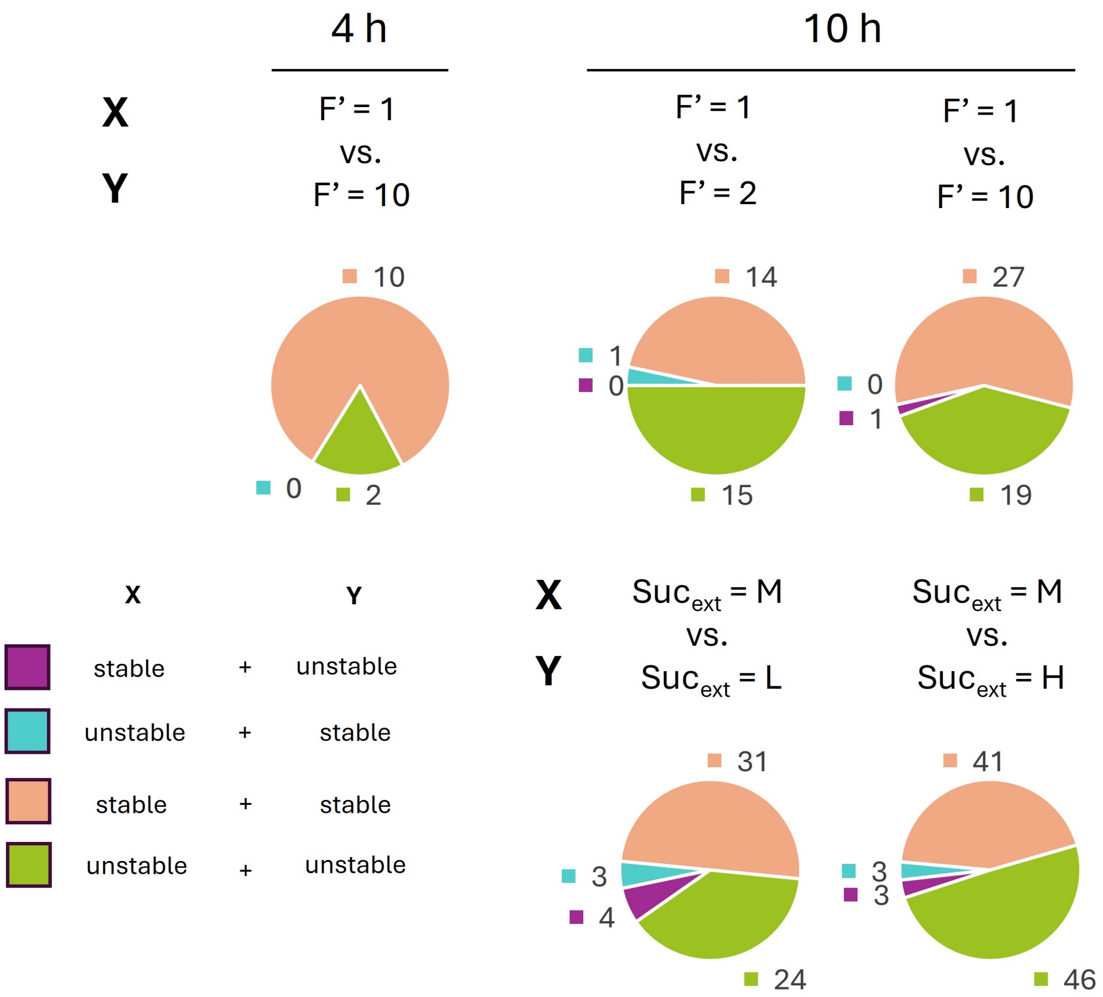

Figure S3: **Stability analysis overview of different F flux sizes and external sucrose concentrations.** A few of the 118 regulation combinations were tested with other fluxes F and other external sucrose concentrations for the 4 h datasets (A) and the 10 h datasets (B). The different tested external sucrose concentrations were 2/3 of the cytosolic sucrose concentration (L), the same as the cytosolic sucrose concentration (M) and 3/2 of the cytosolic sucrose concentration (H).

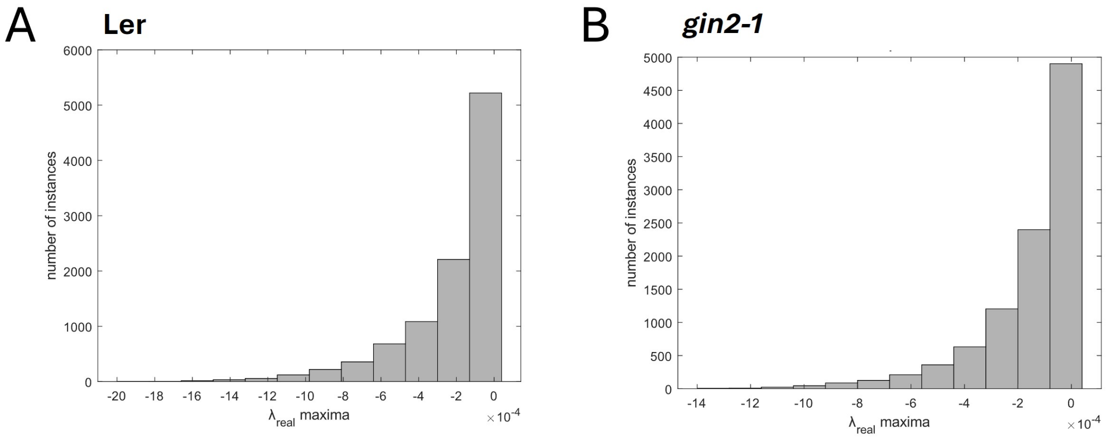

Figure S4: **Histograms of Eigenvalue real part maxima.** Histograms of maximal real part Eigenvalues for 10^6 calculations without regulatory instances with medium external sucrose concentration in (A) wild type *Arabidopsis thaliana* Ler and (B) mutant *gin2-1*.

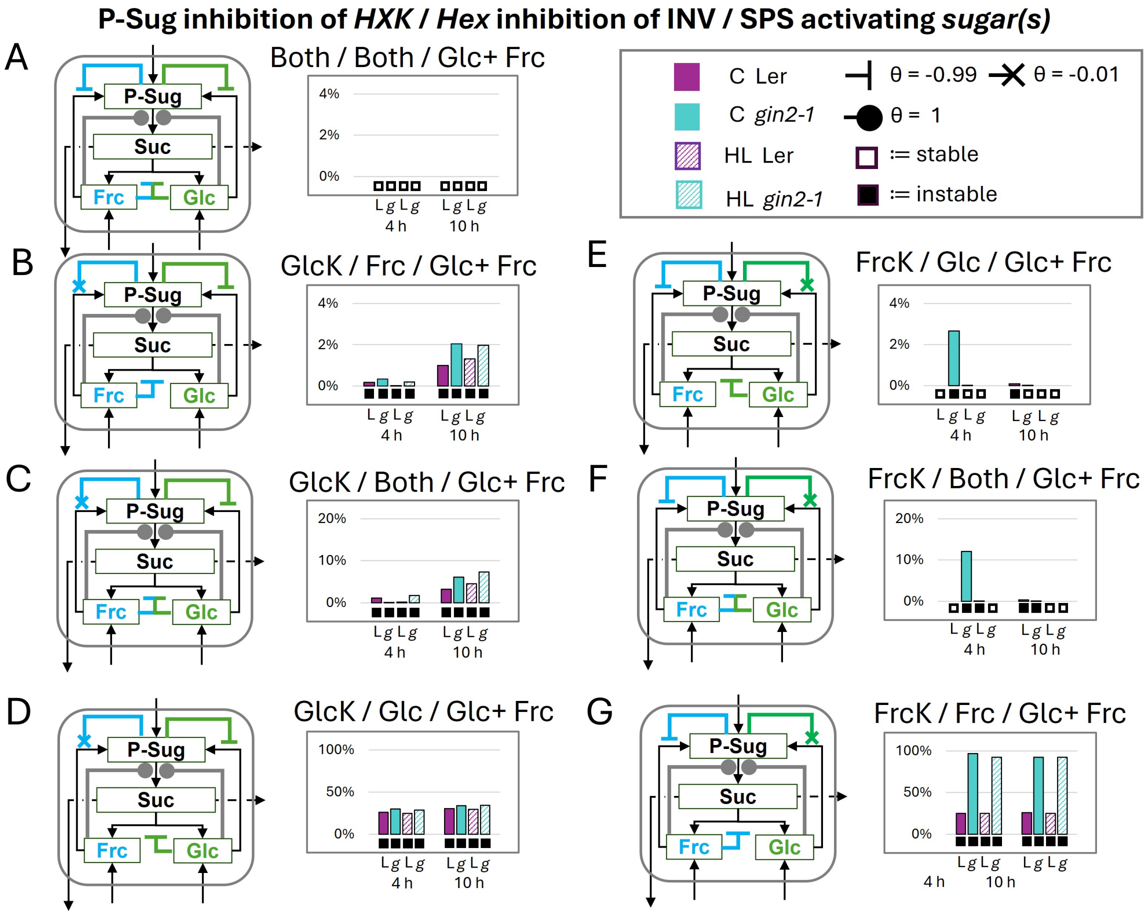

Figure S5: **Influence of additional weak hexokinase inhibitions to prior regulations on the cytosolic sucrose cycle stability.** Stability analysis of full activation of sucrose-phosphate synthase (SPS) by both non-phosphorylated hexoses. In addition to the prior regulations (Fig. 3) the non-inhibited hexokinases were weakly (θ = -0.01) inhibited by P-Sug. The combinations are indicated above each diagram as follows: strong inhibition (θ = -0.99) of P-Sug on which *Hexokinase* / which *Hexose* inhibits cytosolic invertase / which *sugar(s)* activate the SPS. A) GlcK+FrcK/Frc+Glc (B – D) strong glucokinase inhibition (E –G) strong fructokinase inhibition. Note: Here only the cytosolic regulations are depicted, but the model consisted of the whole cell (Fig. 1).
Boxes below diagrams indicate stability (empty box) and instability (black box). L:= Ler (purple, control/high-light:= filled/crosshatched), g:= *gin2-1* (turquoise, control (C)/high-light (HL):= filled/crosshatched), HXK:= hexokinase (GlcK:=glucokinase, FrcK:=fructokinase), INV:= invertase (here cytosolic), Frc:= fructose, Glc:= glucose, P-Sug:= phosphorylated sugars, Suc:= sucrose

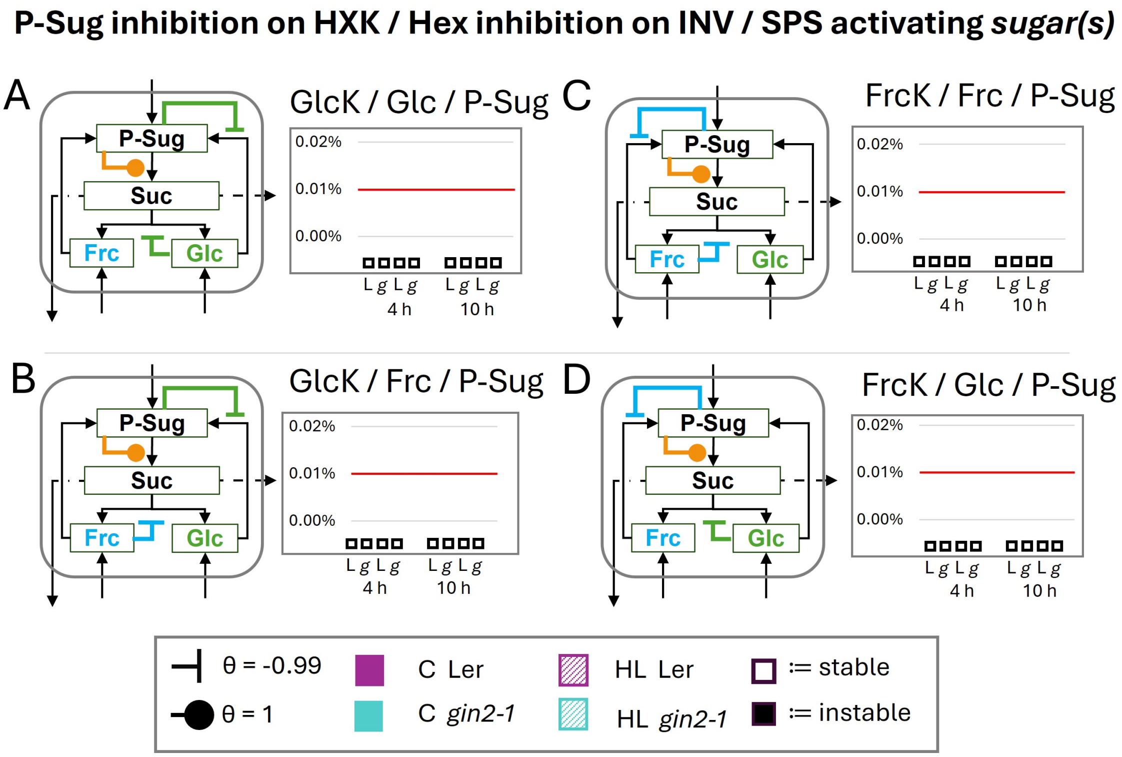

Figure S6: **Influence of single activated sucrose-phosphate synthase (SPS) by phosphorylated sugars on the cytosolic sucrose cycle stability.** Stability analysis of full activation of sucrose-phosphate synthase (SPS) by phosphorylated sugars (P-Sug). The subsequent combinations are indicated above each diagram as follows: inhibition of P-Sug on which *hexokinase* / which *hexose* inhibits cytosolic invertase / which *sugar(s)* activate the SPS. (A - B) glucokinase inhibition (C –D) fructokinase inhibition. Note: Here only the cytosolic regulations are depicted, but the model consisted of the whole cell (Fig. 1).

Boxes below diagrams indicate stability (empty box) and instability (black box). L:= Ler (purple, control/high-light:= filled/crosshatched), g:= *gin2-1* (turquoise, control (C)/high-light (HL):= filled/crosshatched), HXK:= hexokinase (GlcK:=glucokinase, FrcK:=fructokinase), INV:= invertase (here cytosolic), Frc:= fructose, Glc:= glucose, P-Sug:= phosphorylated sugars, Suc:= sucrose; Red line cut-off for instability
